# Identification of Tropomyosin and Its Immunological Properties from Larvae of Cattle Tick, *Boophilus annulatus*

**Published:** 2013

**Authors:** S Nabian, M Taheri, R Mazaheri Nezhad Fard, M Aramoon

**Affiliations:** 1 Department of Parasitology and Research Center of Ticks and Tick Borne Disease, Faculty of Veterinary Medicine, University of Tehran, Tehran, Iran; 2 Rastegar Reference Laboratory, Faculty of Veterinary Medicine, University of Tehran, Tehran, Iran; 3 Arian Hospital, Gonbad Qaboos, Iran

**Keywords:** *Boophilus annulatus*, Tropomyosin, Vaccine

## Abstract

**Background:**

*Boophilus annulatus* is an obligate blood feeder tick that can cause great losses in animals due to anemia and its ability to injure its host skin directly. The aim of this study was identification of cattle humoral immune response to some tick proteins during experimental infestation.

**Methods:**

Immune sera against tick were collected from experimentally infested cattle with ticks. One and two-dimensional electrophoresis and Western blotting methods were used for the detection of immunogenic proteins in larval tick extract and eight of these proteins were identified by MALDITOF and MALDI-TOF-TOF mass spectrometry.

**Results:**

In non-reducing one-dimensional SDS-PAGE, some bounds between 12 to more than 250-kDa appeared. In two-dimensional SDS-PAGE, numerous spot appeared and the identified immunogenic proteins by parallel immunoblotting weighted between 14 and 97 kDa. Amino acid sequences of protein spot with 37-kDa molecular weight had identity to tropomyosin based on Mascot search in NCBI.

**Conclusion:**

Anti tropomyosin antibodies can be induced in experimentally infested hosts with ticks and it seems that tropomyosin can be useful for the development of anti tick vaccines.

## Introduction

*Boophilus annulatus* is an important tick that transmits *Babesia bigemina*, *B. bovis* and *Anaplasma marginale* to cattle. Heavy infestations with the tick cause damages to hides and possibly lead to reduction in the growth rate of cattle (1).

Conventional tick control methods have mainly been based on the use of acaricides; however, the appearance of resistance in tick populations and the presence of chemical residues in nature, meat and milk reveal the need for novel and biological control methods, such as anti tick vaccination (2, 3). Although an anti-tick vaccine may be the most promising control method, its development still depends on the identification and characterization of one or more protective tick antigens (4).

Tropomyosin is an allergenic, actin-binding protein that regulates the actin organization and is a proposed antigen for vaccine development in several species of the parasite (5). Along with the troponin complex, it associates with actin in muscle fibers and regulates muscle contraction by regulating the binding of myosin. Furthermore, actin filament system (cytoskeleton) is required to perform a remarkable array of functions in eukaryotic cells, including cytokinesis, cell motility, contractile force, intracellular transport, cell morphology and cell size. Tropomyosin isoforms have an important role in generation of actin filament functional diversity (6). A few researches have been carried out on the tick proteomics in Iran, so more knowledge on tick proteins and their characterization can be useful for development of tick vaccine.

In the current study, humoral immune responses were investigated in cattle after experimental exposure to *B. annulatus* and some immunogenic proteins identified using 2D-electerophoresis and Western blotting in unfed larval tick extracts.

## Materials and Methods

### Tick collection and rearing

Tick collection and rearing were chosen according to Brown et al. (7). The adult *B*. *annulatus* ticks used in this study were originated from the Band pay of Mazandaran Province (north of Iran) and reared on Here-ford calves maintained in the Large Animal Teaching Hospital, Karaj. Eggs were obtained by maintaining fully engorged females at 28°C and 80% relative humidity for oviposition. Hatched larvae were maintained and grown up in the same condition, in Parasitology Laboratory of the Faculty of Veterinary Medicine, University of Tehran. Larvae were stored at － 70 °C at the 12^th^ day after hatching until use.

### One-dimensional electrophoresis

Initial studies included 12% tris-glycine/SDS gels and running buffers under a non-reducing condition. All separations of tick larvae proteins were performed using a mini gel electrophoresis system (BioRad, USA). Protocols and methods were as described by Lamelli (8). *B. annulatus* unfed larvae were grounded and homogenized in PBS, then 6 µl (10 µg) of the protein were diluted two folds in SDS-PAGE sample buffer and then electrophoresed on gel at 80 V for 90 min. Coomassi and silver stains were used to visualize proteins.

### Two-dimensional electrophoresis

Unfed larva homogenates (0.1 g/3 ml) were prepared in lysis buffer (8 M urea, 2 M thio urea, chaps 4%, biolit 0.2%, 4 mM tris pH = 7). The homogenates were centrifuged at 12000 g at 4°C for 15 min. After separation of supernatants, the protein concentration was measured using Bradford assay. Prior to isoelecteric focusing, 300 µg of protein were loaded onto 7-cm IPG strips (pH = 3–10 NL). The sample was rehydrated overnight followed by a step voltage focusing procedure (250 V, 20 min; 4000 V, 2h; reached to 14000 V, 4 h) (9). Strips were incubated in 10 ml equilibration buffer (50 mM tris, 6 M urea, 2% SDS, 30% glycerol, pH = 8.8) containing 30 mM dithiothretiol for the first 20 min and replaced by equilibration buffer with 135 mM iodoacetamide for another 20 min. Electrophoresis in second dimension of SDS-gel (12%) was carried out in an electrophoresis system (BioRad, USA). Coomassi brilliant blue R-250 was used to visualize proteins.

### Immunoblotting

Immunoblotting was carried out according to original protocols by Wang et al. (10) with some modifications. Larval tick protein extracts were separated in 12% SDS-PAGE gels and transferred to nitrocellulose membranes using a wet system (BioRad, USA) in transfer buffer. The membrane blocked 45 min with PBS containing 2.5% Tween 20 and all the washing steps were carried out with PBS containing 0.05% Tween 20 (3 × 5 min). The membrane was incubated one hour in serum derived from cattle experimentally infested with *B*. *annulatus* larvae (1/50 in PBS containing 0.05% Tween 20) at room temperature; then, washed and incubated with anti-bovine IgG coupled to horse radish peroxidase (1/1000 in PBS) for 30 min at room temperature. After washing, the color development step was performed with diamino benzidine containing H_2_O_2_.

### Mass Spectrometry

Based on the Western blot results, eight of the immunogenic protein spots were excised from parallel 2-D gel stained with Coomassi blue, and subjected to York University in England After staining, numerous spots were detected (Fig. 3). *B. annulatus* larval extract was reacted with collected sera from cattle, which have been for MALDI-TOF and MALDI TOF/TOF mass spectrometry. The spectral data were compared with data annotated to NCBI database using Mascot software.

## Results

### 1-D SDS-PAGE

**Fig. 1:**
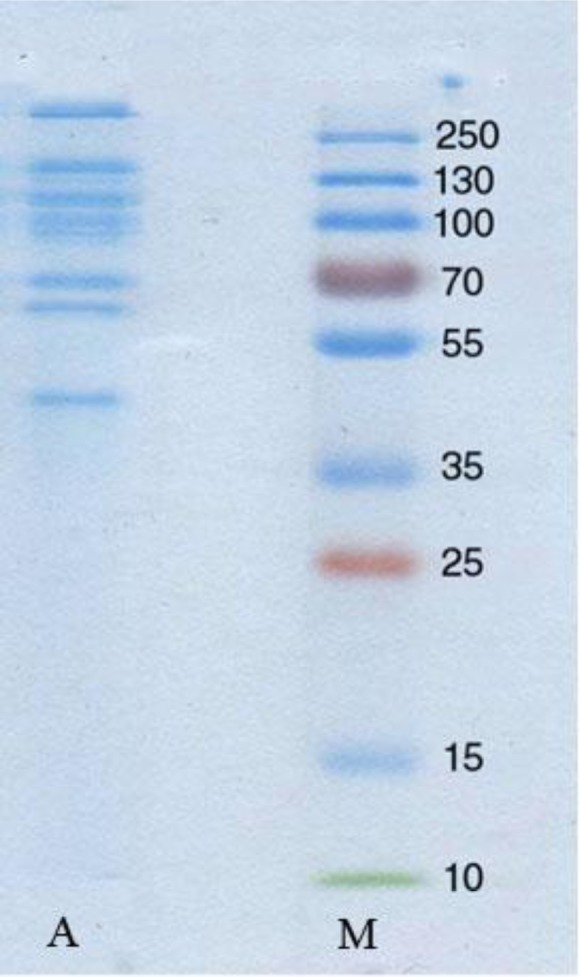
Non reducing SDS-PAGE (12% gel) of *B. annulatus* unfed larval extract (Coomassi blue staining). Lane A-Tick larval extract, Lane M- Molecular weight standards

After electrophoresis under non-reducing conditions, eight sharp bands with molecular weights between 40 and more than 250 (kDa) were detected (Fig. 1).

**Fig. 2:**
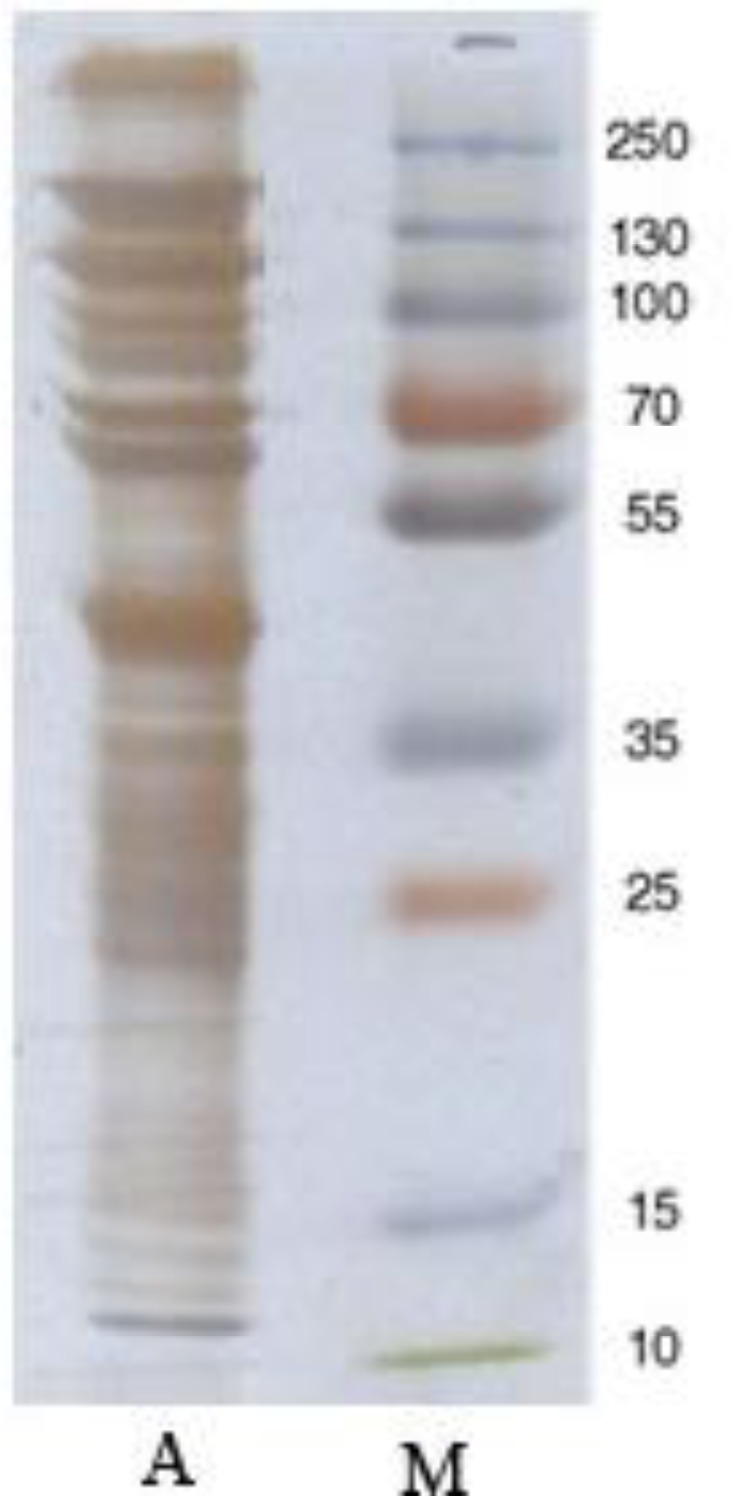
Non-reducing SDS-PAGE (12% gel) of *B. annulatus* larval extract (Silver staining). Lane A-Tick larval extract, Lane M- Molecular weight standards

In non-reducing SDS-PAGE with silver staining, more bands between 12 and more than 250 kDa were detected (Fig. 2).

**Fig. 3:**
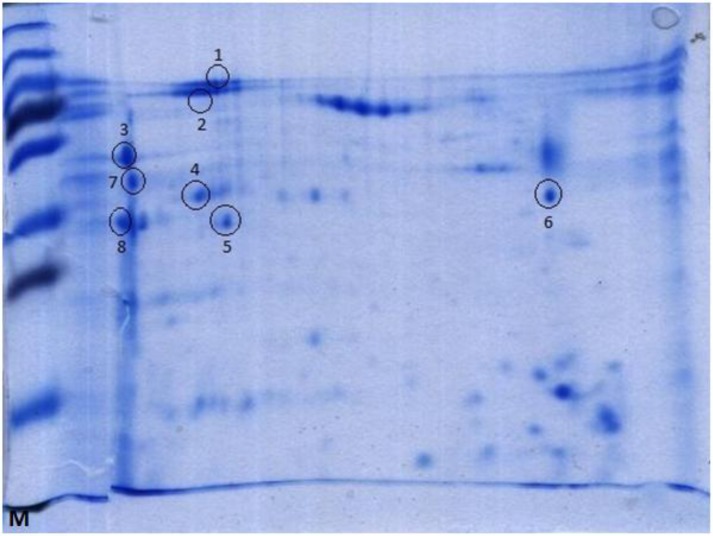
Representative 2-D gel electropherogram of proteins from unfed *B*. *annulatus* larval extract (Coomassi blue staining), M- Molecular weight standards

### 2-D SDS-PAGE and immunoblotting

**Fig. 4:**
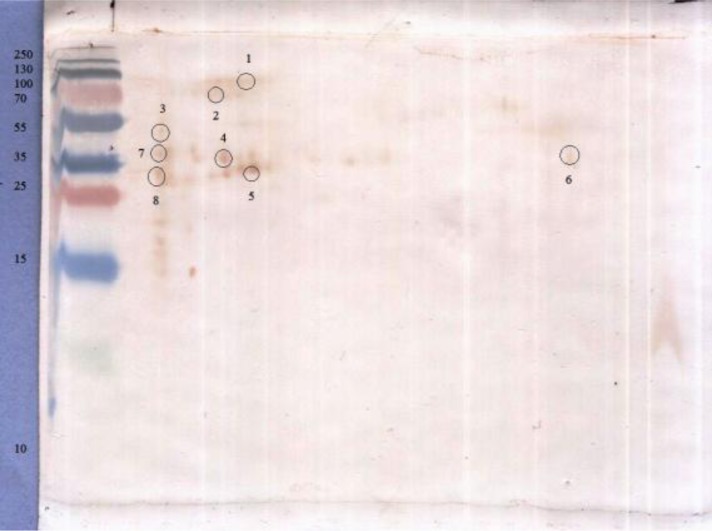
Two-dimensional Western blot analysis (12% gel, IEF pH 3–10) of *B. annulatus* larval extract using immune sera. M- Molecular weight standards

*B. annulatus* larval extract proteins (300 µg) were separated using isoelectric focusing on a non-linear immobilized pH gradient (3–10) and second dimension electrophoresis on a 12% gel. experimentally infested with tick six times. Some of the spots related to Coomassi blue stained 2-D gels were detected as immunogenic spots based on the results of 2-D Western blotting by immune sera. Eight immunogenic spots (nos 1-8), between 14 and 97 kDa, were analyzed by MALDI-TOF and MALDI TOF/TOF mass spectrometry (Fig. 4).

**Fig. 5:**
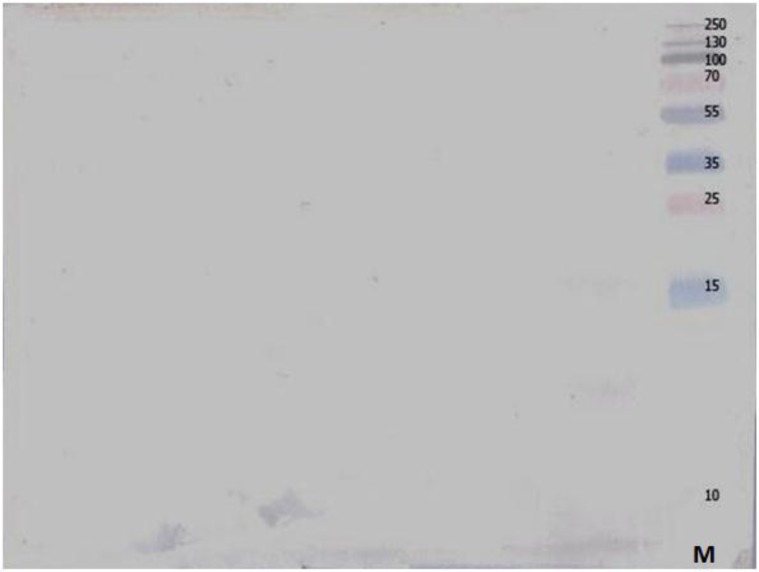
Two-dimensional Western blot analysis (12% gel, IEF pH 3–10) of *B. annulatus* larval extract, using non-immune sera. M- Molecular weight standards

A little or no reactive spots were observed in blots incubated with non-infected cattle sera as control (Fig. 5).

### Mascot Search Results

**Fig. 6:**
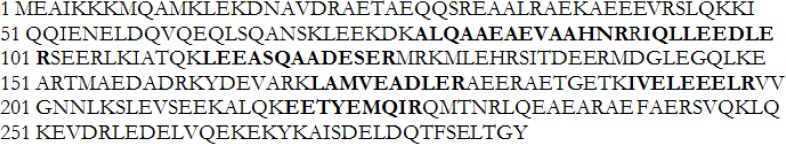
Amino acid sequences of *B*. *micropolus* tropomyosin (matched peptides from current study are shown in Bold)

The MALDI-TOF and MALDI TOF/TOF analysis against the NCBI database restricted to Metazoa provided evidence that spot number six is homologus of *B. micropolus* tropomyosin. Four protein spots (1, 2, 4, 7) were related to vitellogenin. Spot number 5 had a single peptide match to hypothetical protein ISCW001652 (*Ixodes scapularis*). Two protein spots (3, 8) were not identifiable, suggesting that they may be novel molecules. Protein view for spot number six is shown in Fig. 6. Peptide sequence search results using MS/MS for protein number 6 are shown in Table 1.

**Table 1:** Some Proteins matched to subset of peptides sequences obtained from spot number 6 of *B. annulatus* larval extract, using MS-MS (Mascot search)

	Protein ID^a^	Genbank Accession No.	Mass (kDa)^b^	Score^c^	Matches
1	*Boophilus microplus* tropomyosin	gi 42559557	32.982	454	6
2	Unknown tropomyosin *Haemaphysalis qinghaiensis*	gi 148616183	32.893	397	4
3	Tropomyosin invertebrate, putative (*ixodes scapularis*)	gi 241574488	34.694	292	3
4	Tropomyosin *Dermanyssus gallinae*	gi 83308265	32.654	254	3
5	Tropomyosin isoform 9A *Drosophila melanogaster*	gi 158693	34.169	197	2
6	Tropomyosin isoform 33(9c) *Drosophila melanogaster*	gi 158695	56.128	197	2
7	Fast tropomyosin isoform *Homarus americanus*	gi 2660868	32.817	197	2
8	Tropomyosin *Thyrophygus* sp.	gi 40548519	32.807	197	2
9	Tropomyosin invertebrate (*Aedes aegypti*)	gi 157131823	31.039	195	2
10	Tropomyosin II non-muscle isoform *Drosophila melanogaster*	gi 158700	29.405	158	2
11	Hypothetical protein LOAG_08281 Loa Loa	gi 312083436	18.097	107	1
12	Tropomyosin B *Echinococcus granolosus*	gi 168071448	25.782	68	1
13	Tropomyosin *Schistosoma mansoni*	gi 256078892	32.991	68	1

a Protein in the NCBI database to which significant peptide mass matching was observed.

b Predicted mass values determined by database search algorithms, estimated from their amino acid sequences.

c Protein score is derived from ion scores (sum of ion scores). Ions score is -10 Log (*P*), where *P* is the probability that observed match is a random event.

## Discussion

*Boophilus annulatus* is a single-host tick that causes important losses to bovine herds annually. Protective antigens are being investigated in order to develop vaccines to avoid the use of acaricides. Therefore, proteome analysis of *B. annulatus* larvae can help to candidate targets to develop novel control strategies. Two-dimensional electrophoresis is one of the appropriate methods, which can be used for the separation, and quantification of proteins (11). Although 1-D electrophoresis does not have the same resolving capacity, it can be useful in conjunction with 2-D electrophoresis. In this study, for the first time we identified immunogenic proteins in *B.annulatus* by a combination of 2-D electrophoresis and Western blotting. One of the eight spots (with 37 kDa molecular weight) that submitted for MALDI-TOF MS/MS was identified as trpomyosin, which reacted with immune cattle serum. As shown in Table 1, tropomyosin proteins from other invertebrates matched with this protein.

There are multiple tropomyosin isoforms in invertebrates, which suggests the complexity of tropomyosin function. Tropomyosin has a wide distribution in muscle and non-muscle tissues. Vertebrate muscle tropomyosin is a dimer molecule arranged in an in-parallel, inregister coiled coil (12). Sometimes, muscle-type tropomyosin is expressed in non-muscle tissues. Some investigations have suggested that tropomyosin isoform expression is highly regulated during the development and between different cells (13). The amount and isoform type of tropomyosin in actin filaments are important for specializing the actin function (6).

In order to identify functional genes, Zhao et al. (14) constructed cDNA expression library from mRNA extracted from larval *Haemaphysalis qinghaiensis* ticks. They found 11 positive clones from the library through immunoscreening of the library using the polyclonal antibody generated in rabbits with larval tick protein extracts. The amino acid sequences of one of these clones shared 94%, 96% and 80% identities with the tropomyosin mRNAs of *H. longicornis*, *B. micropolus* and *Aleuroglyphus ovatus* ticks, respectively (NCBI/ BLASTIN). They suggested that tro-pomyosin might be a vaccine candidate for *H. qinghaiensis*. Nisbet et al. (5) characterized tropomyosin, its immunogenicity and tissue distribution in poultry red mites. Encoded proteins from cDNA showed 89% and 88% identities to tropomyosins from *B. micropolus* and *Haemaphysalid longicornis* respectively, and 85% identity to the house dust mite tropomyosin. They also showed the mouse antibodies against house dust mite tropomyosin reacted with a band of 38 kDa on Western blots of *D. gallinae* extract, consistent with the molecular masses of acarin tropomyosin. The IgY component of the sera from infested hens in Western blot of *D. gallinae* extract did not bind to tropomyosin, but reacted with a number of mite proteins. Therefore, they concluded that antitropomyosin antibodies were not induced in hens after the natural exposure to mites. Their immunolocalization study of tropomyosin in mites showed a vast distribution of the molecule in mite tissues.

Aki et al. (15) showed that tropomyosin is an allergenic protein and reacts with specific IgE in sera of humans allergic to house dust mite. They have also shown that tropomyosin of the sheep scab mite *Psoroptes ovis* can induce IgE and IgG responses in sheep during infestation (16). Hartmann et al. (17) showed that immunization of jirds with a purified protein (41 kDa tropomyosin-like protein) from the filarial nematode *Acanthocheilonema viteae* caused the reduction of adult worm burden by > 60% and the circulating microfilariae by up to 93%. The role of tropomyosin in host resistance to microfilaria in onchocerciasis was investigated by Rosalind et al. (18) and Folkard et al. (19). They concluded that muscle isoform of tropomyosin from dying or dead microfilaria may induce a humoral response that can cross react with a non-muscle form, which can injure the parasite most likely via antibody-dependent cell toxicity. This phenomenon was supported by the attachment of anti-tropomyosin antibodies to the cuticle of microfilariae, as confirmed by immunoelectron microscopy. Therefore, they suggested that anti-tropomyosin antibodies, in parallel with antibody independent mechanisms, could influence microfilarial densities.

In current study, tropomyosin of *B. annulatus* larval extract migrated with an apparent molecular mass of 37 kDa on SDS-PAGE; however, the predicted size of this isoform was 32.9 KDa. According to Untalan et al. this difference in molecular masses could be attributed to post-translational modifications, multiple protein isoforms and different protein compositions (20). These data were similar to data published by Rosalind et al. (18), who showed a tropomyosin isoform from *Onchocerca vulvulus* with an apparent molecular mass of 42 kDa on SDS-PAGE, but with predicted size of 33.2 KDa. They also suggested that the protein might be post-translationally modified. Moreover, they obtained sequences, which shared 91% identity with tropomyosin from other nematodes.

## Conclusion

Antitropomyosin antibodies are induced in cattle after experimental exposure to ticks and may help to protect against tick infestation; therefore, tropomyosin can be considered as a antigen for anti tick vaccine.
